# Whole-Genome Sequence of *Brucella canis* Strain SVA13, Isolated from an Infected Dog

**DOI:** 10.1128/genomeA.00700-14

**Published:** 2014-07-17

**Authors:** Rene Kaden, Joakim Ågren, Sevinc Ferrari, Martina Lindberg, Stina Bäckman, Tara Wahab

**Affiliations:** a National Veterinary Institute, Uppsala, Sweden; b Swedish Forum for Biopreparedness Diagnostics, Umeå, Uppsala, and Solna, Sweden; c Uppsala University, Department of Medical Sciences, Uppsala, Sweden; d National Food Agency, Uppsala, Sweden; e Swedish Defence Research Agency, Umeå, Sweden; f Public Health Agency of Sweden, Solna, Sweden

## Abstract

An outbreak of canine brucellosis in Sweden was confirmed by the National Veterinary Institute (SVA) in August 2013. The whole genome of the causative agent was sequenced, assembled, and analyzed.

## GENOME ANNOUNCEMENT

*Brucella canis* is a pathogenic facultative intracellular bacterium. The species is assigned to biosafety level 3 due to its low infectious dose, zoonotic potential, and difficulties in treating the disease (1). Sweden is officially free of brucellosis. All human and animal infections are therefore acquired abroad. The current outbreak of canine brucellosis was caused by a male dog that was imported from Spain for breeding. *B. canis* was cultivated from aborted material on selective medium, and the DNA of the grown bacteria was extracted using an EZ1 DNA tissue kit with an EZ-1 extraction robot (Qiagen), according to the manufacturer’s protocol. The libraries for whole-genome sequencing (WGS) were prepared with a Nextera XT sample preparation kit. An Illumina MiSeq platform with a 2 × 300 paired-end run was used for whole-genome sequencing. The single reads were assembled into contigs with Mira 3.9.15 (2). The assembling of the whole sequences was done with SeqMan 8.0.2 and manually using the reference sequence of the *B. canis* type strain ATCC 23365 (accession no. NC_010103 for chromosome 1 and accession no. NC_010104 for chromosome 2). Gegenees software version 2.0 with a threshold of 20% was applied to determine the average nucleotide identity (ANI) (3). Annotation was done by the NCBI Prokaryotic Genome Annotation Pipeline with GeneMarkS+, with the best-placed reference protein set method. Tandem-repeat analysis was realized using Tandem Repeats Finder (4).

The genome of *B. canis* strain SVA13 differs by 0.04% from that of the type strain of the species, *B. canis* ATCC 23365, and the G+C content is 57.24%. The circular chromosomes 1 and 2 consist of 2,106,955 and 1,203,360 bases, respectively. The whole genome contains 3,093 genes and 2,950 coding sequences (CDSs). Three prokaryotic rRNA types, 5S, 16S, and 23S, with a total number of 16 operons and one noncoding RNA (ncRNA), as well as 55 tRNA operons, exist in the genome. In total, there are 57 genes with a frameshift mutation present in the genome. Chromosome 1 contains 60 tandem repeats with a length of ≥8 bases and a copy number of 2 to 13. The 30 tandem repeats that were detected in chromosome 2 had a length of 5 to 264 bases with a copy number of 2 to 11.

### Nucleotide sequence accession numbers.

The whole genome of *B. canis* strain SVA13 was deposited at DDBJ/EMBL/GenBank under accession no. CP007629 and CP007630 for chromosomes 1 and 2, respectively. The version described in this paper belongs to NCBI BioProject PRJNA242400 and NCBI BioSample SAMN02739937.
